# Impact of a Nutrient Formulation on Longitudinal Myelination, Cognition, and Behavior from Birth to 2 Years: A Randomized Clinical Trial

**DOI:** 10.3390/nu15204439

**Published:** 2023-10-19

**Authors:** Nora Schneider, Mickaël Hartweg, Jonathan O’Regan, Jennifer Beauchemin, Leanne Redman, Daniel S. Hsia, Pascal Steiner, Owen Carmichael, Viren D’Sa, Sean Deoni

**Affiliations:** 1 Brain Health, Nestlé Institute of Health Sciences, Nestlé Research, Société des Produits Nestlé S.A., 1010 Lausanne, Switzerland; 2 Biostatistics and Data Management, Clinical Research Unit, Nestlé Research, Société des Produits Nestlé S.A., Vers-chez-les-Blanc, 1000 Lausanne, Switzerland; 3 Nestlé Development Centre Nutrition, Askeaton, Co., RH6 0PA Limerick, Ireland; 4 Advanced Baby Imaging Lab, Hasbro Children’s Hospital, Providence, RI 02903, USA; 5 Pennington Biomedical Research Center, Baton Rouge, LA 70808, USAowen.carmichael@pbrc.edu (O.C.); 6 Department of Pediatrics, Brown University, Providence, RI 02903, USA; 7 Spinn Neuroscience, Mukilteo, WA 98275, USA

**Keywords:** infant nutrition, brain development, myelination, MRI, phospholipids, sphingomyelin

## Abstract

Observation studies suggest differences in myelination in relation to differences in early life nutrition. This two-center randomized controlled trial investigates the effect of a 12-month nutritional intervention on longitudinal changes in myelination, cognition, and behavior. Eighty-one full-term, neurotypical infants were randomized into an investigational (N = 42) or a control group (N = 39), receiving higher versus lower levels of a blend of nutrients. Non-randomized breastfed infants (N = 108) served as a reference group. Main outcomes were myelination (MRI), neurodevelopment (Bayley-III), social-emotional development (ASQ:SE-2), infant and toddler behavior (IBQ-R and TBAQ), and infant sleep (BISQ) during the first 2 years of life. The full analysis set comprised N = 67 infants from the randomized groups, with 81 myelin-sensitive MRI sequences. Significantly higher myelination was observed in the investigational compared to the control group at 6, 12, 18, and 24 months of life, as well as significantly higher gray matter volume at 24 months, a reduced number of night awakenings at 6 months, increased day sleep at 12 months, and reduced social fearfulness at 24 months. The results suggest that brain development may be modifiable with brain- and age-relevant nutritional approaches in healthy infants and young children, which may be foundational for later learning outcomes.

## 1. Introduction

Nutrition plays an important role in brain development and learning, particularly during the period of rapid growth in the first years of life [1,2,3]. Several nutritional components of breast milk have been associated with neurodevelopmental outcomes in infants and young children, including long-chain polyunsaturated fatty acids (LC-PUFA; e.g., docosahexaenoic acid (DHA) and arachidonic acid (ARA)) and micronutrients [4,5]. Further, nutrient deficiencies in iron, vitamin B12, and folate, for example, have been associated with suboptimal brain development [6,7,8]. Nutritional intervention studies investigating individual or combined breast milk nutrients in healthy brain and cognitive development suggest some beneficial effects. LC-PUFA, proteins, and more recently milk fat globule membrane (MFGM) components are amongst the most researched supplementations in human infants. MFGM components include phospholipids, such as phosphatidylcholines, phosphatidylserines, gangliosides, and sphingomyelins, as well as different proteins [9,10]. Recent systematic reviews in healthy infants provide evidence for some positive effects of LC-PUFA (e.g., DHA and arachidonic acid (ARA)) on cognitive functions but report no effect of protein level modifications in infant formulas on cognitive development, and beneficial short-term effects of MFGM components [11,12].

While there are many factors and mechanisms that play a role in the link between early life nutrition and cognitive or behavioral development, de novo myelination has shown both relevance and feasibility in researching that relationship. In past work, we demonstrated the effect of a blend of DHA, ARA, vitamin B12, folic acid, iron, and sphingomyelin (SM) on myelin mechanisms in a primary cell culture model, including an increase in the number, differentiation, and maturation of oligodendrocyte precursor cells [13]. More recently, results from a staged statistical analysis of the first two completed time points in a randomized controlled trial testing the same blend of nutrients in neurotypical infants showed increased brain myelination at 3 and 6 months of life in the investigational group compared to the control group [14]. Here, we extend these findings from this clinical trial to report the longitudinal results of brain, cognitive, and behavioral changes up to 24 months. To the best of our knowledge, this is the first nutritional intervention study in term infants to explore the effect of a blend of nutrients on myelination, a process that is critical for cognitive development and learning [15,16]. Observational data from birth cohorts exploring trajectories of white matter maturation, using myelin water imaging, suggest a general trend of a steep increase in myelin in the first 2 years of life and a shoulder point at around 2 years that is followed by continued but less steep increase in myelin [17].

In this randomized controlled investigational trial, we tested the hypotheses that 12-month supplementation with a blend of DHA, ARA, iron, vitamin B12, folic acid, and sphingomyelin from a uniquely processed whey protein concentrate enriched in alpha-lactalbumin and phospholipids in neurotypical term-born children increases myelination over the first 2 years of life.

## 2. Materials and Methods

### 2.1. Trial Design

This prospective, longitudinal, double-blind, randomized controlled clinical trial took place at three clinical sites in the United States (Rhode Island Hospital, RIH; Pennington Biomedical Research Center, PBRC; and Boston Children’s Hospital, BCH) with a two-parallel-group design and a non-randomized reference group of breastfed infants. Randomization was performed using the Medidata RTSM module (dynamic randomization, second-best probability = 15%) and stratified by site and gender. The intervention period was 12 months, and infants were followed up to 2 years of life with the following schedule: V1 (6 ± 1 weeks of life), V2 (3 months ± 2 weeks), V3 (6 months ± 2 weeks), V4 (9 months ± 2 weeks), V5 (12 months ± 2 weeks), V6 (18 months ± 3 weeks), and V7 (24 months ± 4 weeks). The BCH site was closed before study end due to recruitment issues. The two enrolled infants from that site dropped out.

Recruitment occurred from 2017 to 2020, with the First Subject First Visit (FSFV) in March 2017, the Last Subject First Visit (LSFV) in March 2020, completion of the 12-month intervention period for all randomized participants in February 2021, and the Last Subject Last Visit (LSLV) in February 2022. After completion of the intervention phase for all participants, a staged statistical analysis (SSA) was approved by the Institutional Review Boards and performed for main outcomes of the 3- and 6-month data only. For the SSA, a collaborator at one of the clinical sites and an external contract research organization were unblinded; however, only aggregated information was shared with the sponsor to ensure blinding until trial completion. The SSA results have been reported in our previous publication that describes recruitment, randomization, blinding, data, and biological sample collection [14]. After a parent or guardian provided written informed consent for themselves and for the child at a screening visit, sociodemographic information, medical history, and family history were collected and physical and neurological examinations of the infant were completed. Withdrawal from the study was possible at any point. The Institutional Review Boards at all clinical sites approved and monitored the protocol.

### 2.2. Trial Interventions

Intervention products were bovine-milk-based infant formulas manufactured by Wyeth Nutrition, Askeaton, Ireland, containing a selected blend of nutrients naturally occurring in breast milk and hypothesized to promote myelination based on observational and in vitro findings. Both intervention products included the same organoleptic and sensory characteristics to secure blinding and contained the same blend of nutrients but at different levels. The investigational products contained higher levels of DHA, ARA, iron (fortified through ferrous sulfate heptahydrate), folic acid and vitamin B12 (fortified through cyanocobalamine), and an alpha-lactalbumin-enriched whey protein concentrate with higher levels of sphingomyelin and phospholipids than the control product due to the ingredients’ unique manufacturing process. Nutrient levels in the investigational products as well as in breast milk of the study population have been reported previously [14]. The levels were in line with regulatory requirements for infant nutrition products and are in most cases mandated for inclusion as individual compounds in routine-use infant formulas for healthy infants. The infant formulas were administered orally and ad libitum by the caretaker based on a response-feeding approach. The sponsor adhered to the World Health Organization code supporting exclusive breastfeeding for at least the first 6 months of life. The study team did not influence the parental feeding choice at any time. A lactation consultant was freely available to mothers in the breastfeeding group to support and advise mothers regarding lactation.

### 2.3. Trial Outcomes

Main outcomes were longitudinal myelin content measures from MRI-based myelin water imaging [9,18,19]. Additional, secondary outcomes included brain volumetry (i.e., total brain and total white and gray matter volumes), brain microstructure (i.e., fractional anisotropy and mean, axial, and radial diffusivities), myelin g-ratio, and structural and functional connectivity, as well as cognitive, social-emotional, sleep, and behavioral development assessments. Exploratory trial outcomes were a priori considered for descriptive reporting only to limit the inflation of the family-wise error rate. These included measures of child physical growth, body composition, physical activity, and nutrient intake, as well as maternal measures of postnatal depression, intellectual ability estimate, and parenting stress.

### 2.4. MRI Assessment

#### 2.4.1. MRI Acquisition Protocol

Neuroimaging was performed using Siemens (RIH) and GE (PBRC) 3T MRI scanners during natural and non-sedated sleep (daytime nap or nighttime sleep) [20] at V2 (3 months), V3 (6 months), V5 (12 months), V6 (18 months), and V7 (24 months). The multimodal neuroimaging protocol consisted of mcDESPOT multi-component relaxometry imaging (myelin water imaging) to assess brain myelination, high-resolution anatomical T1-weighted MP-RAGE acquisition for volumetric and morphometry analysis, multiple b-shell diffusion-weighted images to examine the tissue microstructure and architecture, and resting-state functional MRI (rsfMRI) to assess functional connectivity (Supplementary Table S1). Data were collected using acoustically de-rated sequences in combination with passive noise-cancellation measures (e.g., sound-insulating foam, ear plugs, and protectors) to minimize sound disruption to the sleeping infants. Infants were further swaddled in a MedVac pediatric immobilizer to help minimize child motion. Throughout scanning, the child was visually monitored for movement, with scanning paused at signs of the child waking or moving. Where possible (i.e., the infant was still asleep), images with motion artifacts were repeated.

#### 2.4.2. MRI Analysis

Following data acquisition, the mcDESPOT and T1-weighted MP-RAGE images were visually examined using SCLD for motion artifacts and, if necessary, excluded from analyses.

From the mcDESPOT data, voxel-wise T1, T2, and myelin water fraction (MWF) measures were estimated using an established processing pipeline that includes linear registration to account for subtle motion between the 26 individual acquisitions, removal of non-brain signals (skull stripping), correction for main and transmit magnetic field (B0 and B1) inhomogeneities, and fitting of single- and multi-component tissue relaxation models using stochastic region contraction. The quantitative T1, T2, and MWF images were then non-linearly aligned to a custom pediatric template in MNI space following a previously described multi-step, multi-scale registration approach using Advanced Normalization Tools (ANTS v2.1 toolbox). Briefly, the high-flip-angle T1-weighted image from the mcDESPOT acquisition was non-linearly aligned to the most appropriate existing age-matched template (constructed for 3, 6, 12, 18, and 24 months) and the transformation applied to the quantitative images. The age-aligned images were then aligned to a single overall template using pre-computed transformations between the age and the study template. Once aligned, mean regional T1, T2, and MWF values were calculated for each child and time point in six a priori (cerebellum; frontal and parietal lobes; and corpus callosum (CC) body, genu, and splenium) and two post hoc (occipital and temporal lobe) white matter (WM) regions of interest (ROIs). In addition, mean values were also calculated for 174 anatomically localized regions throughout the brain, with functional relevance identified through prior analysis.

Anatomical volumetric data were derived from the high-resolution T1-weighted MP-RAGE data. Whole-brain WM, gray matter (GM), cerebellum, and CC volumes were calculated using an atlas-matching approach in which tissue masks in MNI space were aligned to each participant’s image using the inverse of the image transformations calculated in the mcDESPOT processing, thresholded at 0.95, and the volume calculated.

Diffusion tensor and neurite orientation and dispersion density imaging (DTI and NODDI-DTI) processing were performed using the FSL Diffusion Toolbox and the NODDI MATLAB Toolbox, respectively. DTI analysis included eddy current correction, inter-image motion estimation and outlier detection, re-orientation of the b-matrix, and voxel-wise calculation of tensor parameters, including mean, axial, and radial diffusivities (MD, AD, and RD) and fraction anisotropy (FA). For the NODDI analysis, the multi-parameter model was fit to the eddy-current-corrected diffusion-weighted images, providing measures of the orientation dispersion index (ODI) as well as the volume fraction of the intra-cellular or restricted diffusion compartment (VIC) and the volume fraction of an isotropic diffusion compartment (VISO). All diffusion-related parameter images were then aligned to our study template in MNI space using the same multi-step registration approach with the b0 reference image for each child and time point. Once aligned, the mcDESPOT and NODDI parameter images were combined to calculate the myelin g-ratio, a measure of the thickness of the myelin sheath relative to the axon diameter. Mean regional values for all parameters were then calculated for the cerebellum and frontal, parietal, occipital, and temporal lobe WM; the CC body, genu, and splenium WM; and the 174 functionally relevant areas.

Functional connectivity processing was performed for infants 12 months of age and older using the CONN Toolbox (v21). Analysis was restricted to older infants to ensure appropriate contrast within their T1-weighted anatomical images needed for cortical alignment and segmentation.

#### 2.4.3. Multi-Site Harmonization

Matched acquisition protocols were followed across the two imaging centers using custom-coded sequences to help ensure measurement comparability. To assess inter-site consistency of the primary imaging outcomes (relaxometry estimates) across the two imaging centers, a harmonization study was performed using both human “traveling heads” and a commercial quantitative phantom (Essential System phantom from CaliberMRI, which provides 42 NIST-traceable T1 (20–1900 ms), T2 (5–550 ms), and proton density (5–100%) contrast spheres). Matched in vivo and phantom data were acquired at each center with a 1-week inter-visit interval. T1 and T2 measures were calculated for each phantom sphere and throughout the brain. Bland–Altman plots were then calculated for the phantom T1 and T2 results, and a voxel-wise paired t-test was performed between each set of in vivo data collected at the two sites.

### 2.5. Cognitive and Behavioral Assessment

#### 2.5.1. Endpoints for Inference Statistics

Cognitive development was assessed at 6, 12, and 24 months by trained assessors using the Bayley Scales of Infant and Toddler Development, third edition (Bayley-III) [21]. The Bayley-III is a standardized measure to assess development in children between 1 month and 42 months of age in 5 major domains: adaptive behavior, cognition, language, motor development, and social-emotional development. The Bayley-III has good internal consistency (cognitive alpha > 0.79; language alpha > 0.82; motor alpha > 0.86) and good test–retest reliability (cognitive gamma > 0.75; language gamma > 0.69; motor gamma > 0.79) across all composites (Bayley, 2005). The 2 parent-report-based scales (social-emotional and adaptive behavior) were not included. Efficacy endpoints for the clinical trial were the composite scores for motor, language, and cognition.

Social-emotional development was assessed at 3, 6, 12, 18, and 24 months using the Ages & Stages Questionnaires: Social-Emotional, second edition (ASQ:SE-2). The ASQ:SE-2 is a validated parent-rated questionnaire measuring social and emotional development in young children based on 7 subscales: self-regulation, compliance, social communication, adaptive functioning, autonomy, affect, and interaction with people. The reported validity is 84%, and the test-retest reliability is 89% (http://agesandstages.com/products-services/asqse-2/; accessed on 1 March 2017). The efficacy endpoint for the clinical trial was the total score.

Infant and child sleep was assessed at 3, 6, 12, 18, and 24 months of life using the BISQ [22]. The BISQ is a parent-reported brief infant sleep-screening tool capturing the nocturnal sleep duration (between the hours of 7 p.m. and 7 a.m.), daytime sleep duration (between the hours of 7 a.m. and 7 p.m.), the number of night awakenings, duration of wakefulness during the night hours (10 p.m. to 6 a.m.), nocturnal sleep onset time (the clock time at which the child falls asleep for the night), settling time (latency to falling asleep for the night), the method of falling asleep, the location of sleep, and the preferred body position. Sleep problems were rated on a 3-point scale. The BISQ has good, demonstrated test–retest reliability and validity and is applicable for infants and young children 0–3 years of age. Outcome measures for the clinical trial were night sleep (hrs:min), day sleep (hrs:min), total sleep, and the number of night awakenings.

Infant behavior was assessed at 3, 6, and 12 months using the Infant Behavior Questionnaire–revised (IBQ-R) short form [23]. It is a parent-reported measure of infant temperament consisting of 91 items and 15 scales: activity level, distress to limitations, approach, fear, duration of orienting, smile and laughter, vocal reactivity, sadness, perceptual sensitivity, high-intensity pleasure, low-intensity pleasure, cuddliness, soothability, falling reactivity/rate of recovery from distress, and attentional shifting. Parents are asked to rate the frequency of their infant’s temperament-related behaviors observed over the past week on a 7-point Likert scale (never, very rarely, less than half the time, half the time, more than half the time, almost always, always). The measure was developed for parents of children between 3 and 12 months of age. Outcome measures for the clinical trial were mean values for each scale.

At 18 and 24 months, toddler behavior was assessed using the Toddler Behavior Assessment Questionnaire (TBAQ) [24]. It examines temperament-related behavior in 16–36-month-old children and contains 120 items and 11 scales: activity level, perceptual sensitivity, inhibitory control, soothability, appropriate attentional allocation, sadness, object fear, anger, social fear, pleasure, and interest. Answers are given on a 7-point Likert scale (never, very rarely, less than half the time, half the time, more than half the time, almost always, always). Outcome measures for the clinical trial were mean values for each scale.

Two tablet-based, self-constructed performance tasks were administered at 18 and 24 months to assess early learning parameters: (1) Early learning was explored using an object reversal learning task for infants (ORTi), an age-adapted task based on the principles of the probabilistic object reversal task (pORT) [25,26]. The task includes a dog searching for a bone that is hidden under 1 of 3 objects (stimuli). The aim of the task is to find as many bones as possible, with only 1 of the 3 stimuli being rewarded at a time. When the child identifies the right object hiding the bone, they receive positive feedback (a picture of the dog with the bone) and are expected to follow this strategy for as long as it is successful. The paradigm shifts without announcement such that the bone is hidden somewhere else. Now the child should reverse the learned stimuli and strategy and start looking for the new correct stimuli that hides the bone and to follow the new strategy until the next reversal. The task consists of a total of 30 trials. The learning criterion (LC) is reached when the correct stimulus is selected 5 consecutive times, and the bone is randomly reassigned to one of the other two stimuli without announcement. Perseverative errors (PE) are counted when the paradigm shifts, but the child continues selecting the previously rewarded stimulus for more than one trial. A practice trial is provided prior to the task. Efficacy endpoints for the clinical trial included correct hits, total errors/wrong trials, PE after strategy change, the number of sets achieved (LC reached), and trials to reach the LC. (2) Information processing was assessed using a reaction time task (TapTap—iPad based task developed by the senior investigation team—Hasbro Children’s Hospital, 02903 Providence, RI, USA). The child was instructed to tap on a stimulus as soon as it appeared on the screen, with the stimulus appearing in different locations. The child was rewarded for tapping on the stimulus with an animal noise (dog barking). Efficacy endpoints for the clinical trial were time in milliseconds (speed) and the number of correct taps (accuracy). In another condition of the same task, the child was told not to tap on an alternative stimulus that appeared in various locations. The child was rewarded for tapping on the stimulus with an animal noise (dog barking) but not for the stimulus for which the child was told to inhibit the response. Efficacy endpoints for the clinical trial were time in milliseconds (speed), the number of correct taps (accuracy), and the number of inhibitory errors (accuracy).

#### 2.5.2. A Priori Defined Endpoints for Descriptive Statistics

Physical growth (safety assessment) was assessed at each visit. Safety endpoints for the clinical trial were weight (kg), length (cm), head circumference (cm), and corresponding percentiles. The infant body composition was assessed via air displacement plethysmography (PeaPod—COSMED S.R.L. Viale Bruno Buozzi 77, 00197 Rome (Italy)) and described by the total weight, fat, and fat-free mass measured at baseline, 6 weeks, 3 and 6 months, and at 9 months only if the baby’s weight was less than or equal to 17 lb or 7.71 kg and the height was less than or equal to 24.8 inches, or 63.5 cm.

Child activity level was assessed using an activity tracker (FitBit Zip^®^) at 6, 9, 12, 18, and 24 months. Parents were asked to place the device on the diaper on 3 days of the week, starting when the child wakes up from their night’s sleep and to be kept on all through the day until the child goes to bed for the night. Outcome measures for the clinical trial were sedentary (minutes), lightly active, fairly active, and very active levels (minutes).

Maternal postnatal depression was assessed at screening for mothers, if this visit occurred prior to delivery, at 6 weeks and at 3 months postpartum using the Edinburgh Postnatal Depression Scale (EPDS) [27]. The EPDS aims at identifying postnatal depressive disorders in postnatal women. It is a 10-item self-report questionnaire with 4 response options per item. The cut-off score for a possible depression is <10. Outcome measures for the clinical trial were the raw score.

Parenting stress was assessed at 6 weeks and 6, 18, and 24 months of infant age using the Parenting Stress Index, Fourth Edition Short Form (PSI-4-SF). The PSI is a self-report questionnaire to identify potentially dysfunctional parent–child dyads. It consists of 36 items that are combined into 3 scales: parental distress, difficult child characteristics, and dysfunctional parent–child interaction. The questionnaire can be used with parents of children from 1 month to 12 years of age. Outcome measures for the clinical trial were the total score and scores of the 3 subscales.

The maternal IQ estimate was assessed at maternal screening visit 1 (SV1) using the Wechsler Abbreviated Scale of Intelligence, second edition (WASI-II). The four-subtest form of the standardized cognitive ability test consists of the four subscales: vocabulary, similarities, block design, and matrix reasoning. The test is administered by qualified staff and is applicable for children and adults 6 to 89 years of age. The test has demonstrated reliability and validity. Outcome measures for the clinical trial were the full-scale IQ.

Nutritional intake was assessed at 6, 9, 12, 18, and 24 months. Parents were asked to record their baby’s food intake and dietary supplement use for 3 days on a food record collection booklet before defined visits. The booklet was distributed at the previous visit and included food record forms for reporting food intake and dietary supplement use for 3 days, along with instructions for completing the forms. The food records were sent to the University of Minnesota Nutrition Coordinating Center for entry into the Nutrition Data System for Research (NDSR) nutrient and food group intakes. Outcome measures for dietary intake components of the clinical trial were intake of nutrients (macro- and micro-nutrients), nutrient ratios, and food group consumption by age.

### 2.6. Sample Size Justification

A sample size of *n* = 64 infants who complete the trial per intervention group was estimated to be required to cover a mean MWF difference between groups of ≥0.005 (standard deviation (SD) = 0.010) at 2 years (power of 80%, type I error rate at 5%). Due to the operational constraints at the clinical sites during the COVID-19 pandemic, recruitment was stopped at 81 infants in the randomized arms and 108 infants in the breastfed arm.

### 2.7. Statistical Analysis 

All efficacy analyses were conducted according to randomized assignment (ITT principle). Both intervention groups were compared by visit using descriptive statistics, multivariable mixed models corrected for the stratification factors sex and site, and visits, allocated arms, and interactions between visits and allocated arms (or non-parametric Wilcoxon test in the case of failure of the mixed models) (post hoc analyses). Parameters for longitudinal analyses were estimated using either the non-linear mixed model (Gompertz) or the linear mixed model for repeated measurements (MMRM) corrected for individuals’ chronological age (log-transformed, when necessary) and stratification factors. *p*-Values were used to indicate potentially interesting results and were not corrected for multiplicity (compared to the threshold of 0.05). Missing data were considered as missing at random.

Analyses were performed to compare the non-randomized breastfed reference group with the two randomized arms, providing the descriptive statistics and inferential analyses conducted (superiority, two-sided) using mixed models (linear and non-linear) corrected for the stratification factors, visits, allocated arms, and interactions between visits and allocated arms and weighted by the estimated inverse propensity score (PS) of being breastfed (1/PS for breastfed infants and 1/(1-PS) for formula-fed infants). The score was calculated with a logistic regression on breastfed/not breastfed explained using the baseline covariates maternal marital status, family income, maternal full-scale IQ estimate (WASI-II), number of siblings, highest level of education (mother, father, or any adult living with the child), father living with child, and maternal depression score (EPDS) at 6 weeks postpartum. When mixed models were not appropriate even after data log transformation (residues not following a normal distribution or failure to converge), a non-parametrical Wilcoxon test was applied disregarding the propensity score adjustment.

Associations between the MRI outcomes and clinical outcomes were listed as exploratory endpoints and evaluated using Spearman correlation (adjusted for the false discovery rate with the Benjamini–Hochberg method applied on all *p*-values from the Spearman correlation tests). Associations were further investigated by fitting a linear regression adjusted for stratification factors between the clinical outcome and the MRI parameter as a covariate. All statistical analyses were conducted in SAS^®^ 9.4.

## 3. Results

### 3.1. Participants

**Figure 1:**
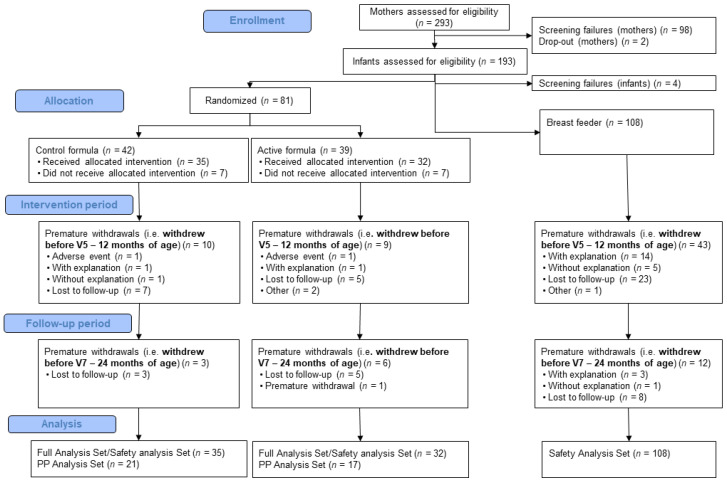
Flow diagram of participants included in the study. Children were randomized into one of two intervention groups, either receiving a blend of docosahexaenoic acid, arachidonic acid, iron, vitamin B12, folic acid, and sphingomyelin from a uniquely processed whey protein concentrate enriched in alpha-lactalbumin and phospholipids or receiving a control formulation. Non-randomized breastfed children served as a natural reference group.

A total of 189 infants were enrolled, of which 81 (42.9%) were randomized into the two intervention groups and 108 (57.1%) participated in the breastfeeding reference group. Compared to our previous publication of SSA results [14], one additional study participant in the control group had data entered for product start in the final database that had not been recorded in the previous database used for the SSA. The mean infant age at enrollment was 29.9 (±7.45) days. Baseline characteristics were similar between intervention groups (Table 1). The full analysis set comprised 175 children, with 67 children in the randomized groups and 108 breastfed children (Figure 1). Reasons for premature withdrawal were evenly distributed between the randomized groups. Feeding characteristics of the study population are reported in Supplementary Table S2.

### 3.2. Product Compliance for Intervention

No major imbalance in the formula feeding frequency was detected between the randomized groups. In both groups, the feeding frequency reduced from 8 feeds per days at 6 weeks postpartum to 3 feeds per day at 12 months of age. Feeding practice was predominantly reported by caretakers as “on demand” and with “no encouragement to finish a bottle”. Few infants had at least one interruption reported per visit (<9% of the infants across visits). Feeding information for study groups is reported in Supplementary Table S2.

### 3.3. Neuroimaging Results

The final dataset for the interventional groups included 99 volumetric datapoints for randomized infants, 81 mcDESPOT relaxometry and myelin datasets, and 39 diffusion datasets.

#### 3.3.1. Harmonization Study

**Figure 2:**
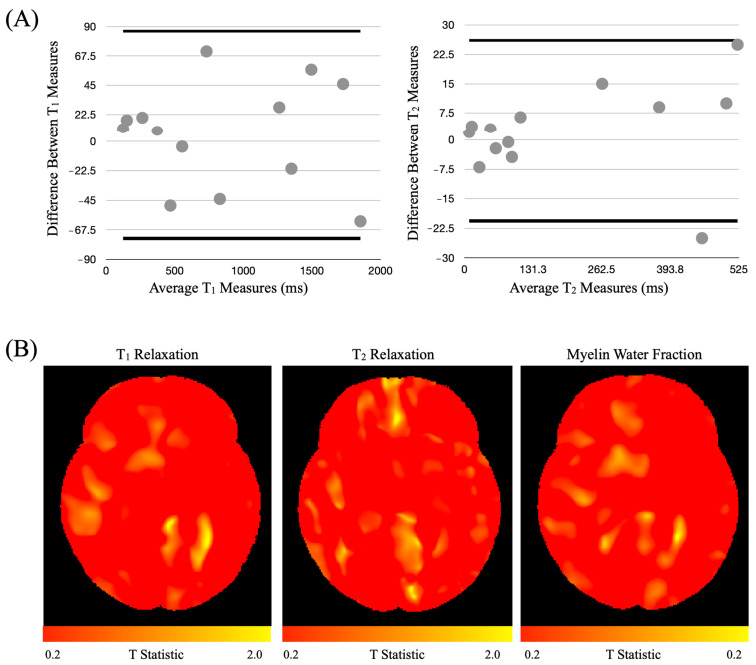
Harmonization study. (**A**) Bland–Altman plot of phantom T1 (**left**) and T2 (**right**) measures revealing no significant biases by site. (**B**) Voxel-wise paired t-tests of in vivo data collected at each site showing significant overlap in values.

Both phantom and in vivo human measures showed strong consistency across the two sites, with correlation coefficient >0.9 in the phantom and >0.8 in the human measures. Bland–Altman plots for the phantom measures are shown in Figure 2A. Results from a voxel-wise paired t-test between the in vivo measures further revealed no significant tissue differences, even when not controlling for multiple comparisons (T-statistic maps shown in Figure 2B).

#### 3.3.2. Myelination (Main Outcome)

**Figure 3:**
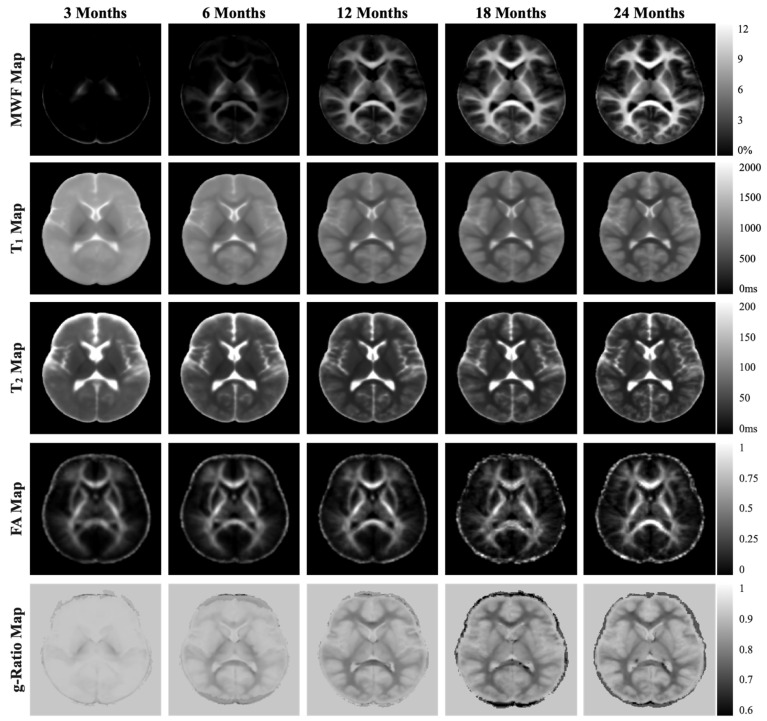
Brain image evolution pooling all study participants from intervention and breastfeeding reference groups across study visits (3, 6, 12, 18, 24 months) for myelin water fraction (MWF), T1, T2, fractional anisotropy (FA), and g-ratio.

Brain myelin development (including myelin water fraction, T1, T2, g-tatio, and FA) at the 3-, 6-, 12-, 18-, and 24-month time points are shown in Figure 3.

Non-linear (Gompertz) longitudinal modeling of the MWF in the predefined ROIs showed no statistically significant differences between the investigational and the control group but indicated a non-significant earlier increase (beta difference = −2.746, 95% CI −8.895 to +3.402), a steeper slope (gamma difference = +1.866, 95% CI −27.375 to +31.108), and a higher plateau (eta difference = +5.470, 95% CI −11.467 to +22.406) in the investigational group.

**Figure 4:**
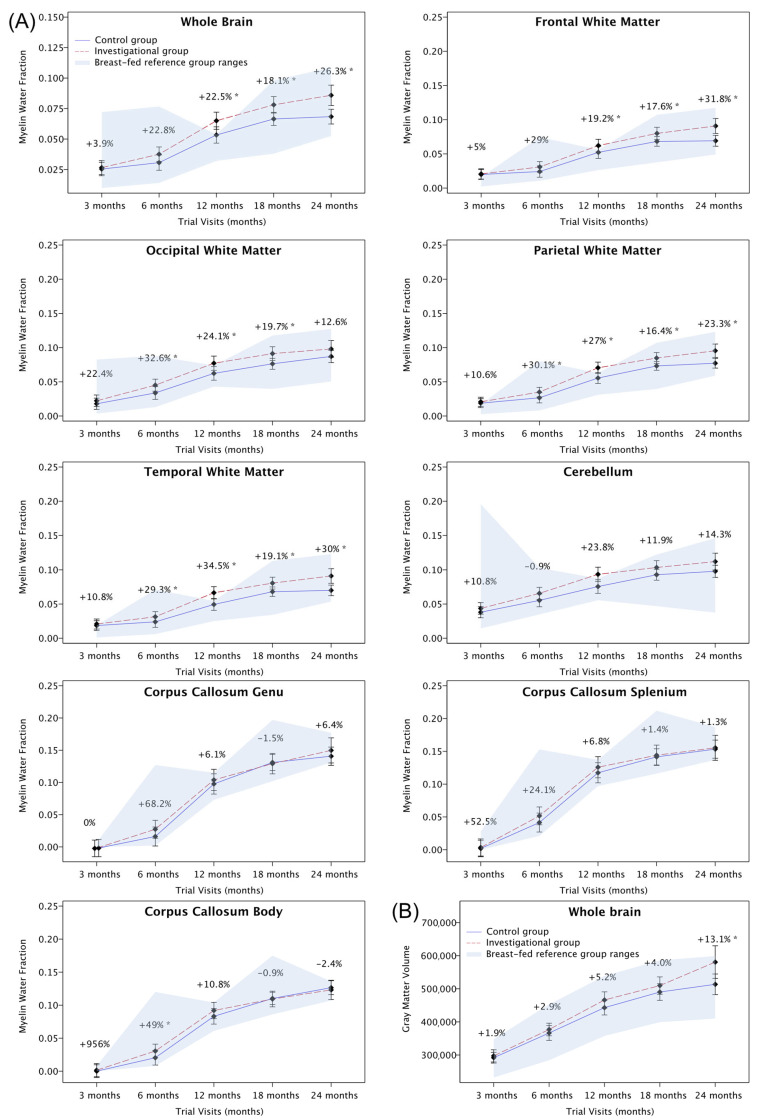
Intervention group effects for MRI-derived myelin and gray matter outcomes based on linear mixed model. (**A**) Whole-brain and regions of interest for myelination. (**B**) Whole-brain gray matter volume. The breastfed reference group ranges show minimum to maximum values. The diamonds connected by lines indicate least square mean estimates with 95% confidence intervals from linear mixed model analyses, adjusted for site and gender. The % values indicate the estimated change between investigational and control groups using least square mean point estimates. * *p* ≤ 0.05.

**Figure 5:**
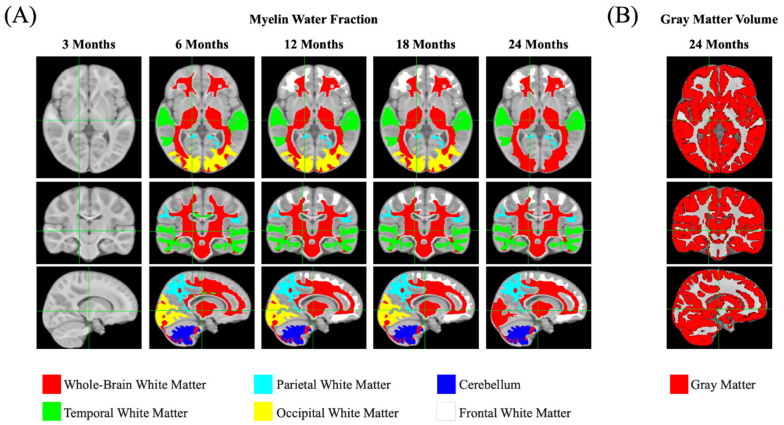
Identified brain regions of interest with significant group differences (in color, *p* ≤ 0.05) between investigational and control groups for myelination (**A**) and gray matter volume (**B**). These predefined regions of interest were analyzed using visit-wise cross-sectional ANCOVAs, adjusted for gender and site.

The limited number of MRI scans in our study led to large confidence intervals on the parameters of the planned non-linear longitudinal modeling. This limitation was addressed with mixed model analyses including visits as a categorical confounder (post hoc). The observed effect size in our study was higher than anticipated (+0.018 versus +0.005), resulting in significantly higher levels of myelin for the investigational versus the control group, despite the smaller than anticipated sample size. Effects were found for the whole-brain MWF at 6 months (+0.007, 95% CI [+0.0012; +0.0126]; *p* = 0.018), 12 months (+0.012, 95% CI [+0.0045; +0.0189]; *p* = 0.002), 18 months (+0.012, 95% CI [+0.0052; +0.018]; *p* ≤ 0.001), and 24 months (+0.018, 95% CI [+0.0086; +0.0264]; *p* ≤ 0.001) (Figure 4). An increased MWF was furthermore found within the cerebellar, frontal, occipital, parietal, and temporal WM. No statistically significant difference was shown for the CC body, genu, and splenium (Table 2, Figure 5). The post hoc analyses may have led to an inflation in the family-wise error rate.

Log-linear longitudinal modeling showed a statistically significant T1 slope difference in the CC splenium for the investigational group (CC splenium slope difference: +76 ms/log(year), 95% CI [0.8; 153]; *p* =0.048). No significant group differences were detected for the g-ratio (whole-brain slope difference: −0.004 mm/mm/log(year), 95% CI [−0.033; +0.025]; *p* = 0.700), T1 (whole-brain slope difference: +1.79 ms/log(year), 95% CI [−45.7; 49.2]; *p* = 0.940), and T2 (whole-brain slope difference: −2.45 ms/log(year), 95% CI [−7.6; +2.7]; *p* = 0.350).

**Figure 6:**
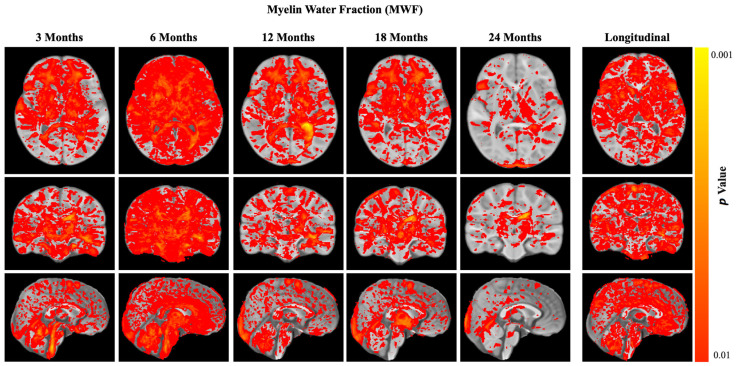
The *p*-value map of age-wise and longitudinal myelin (MWF) differences between investigational and control groups across 174 anatomically relevant areas. Analyses were performed using visit-wise cross-sectional ANCOVAs, adjusted for gender and site.

In addition to the a priori defined brain regions, we also assessed MWF differences (age-wise and longitudinally) across 174 anatomically relevant areas. Results of this post hoc analyses are shown in Figure 6 and revealed widespread increases in myelin water content throughout the brain at 3, 6, 12, and 18 months, including frontal, temporal, and parietal white matter; the cerebellum; the CC; the thalamus; and the brainstem. At 24 months, results appeared to become more central and focused on major white matter regions, including the corpus callosum and thalamic radiations and motor tracts. From the longitudinal data, we observed significant increases in the rate of myelination throughout the brain, including frontal, parietal, temporal, and occipital brain regions; the CC; motor pathways; and frontal cortical gray matter.

For the reference group, longitudinal analyses adjusted for the described propensity score indicated no statistically significant differences between the investigational and the non-randomized breastfed reference group for the MWF and T1. A significant slope difference between the investigational and the breastfed reference group was seen for the CC body T2 (LSMean: −5 ms/year in breastfed reference, *p* = 0.048).

#### 3.3.3. Gray and White Matter Volume (Secondary Outcomes)

Log-linear longitudinal modeling of GM volume showed no statistically significant differences between investigational and control groups (slope difference: +9.6 cm^3^/log(year), 95% CI [−2.6; +21.7]; *p* = 0.120). Mixed model (post hoc) analyses showed significant GM volume differences in favor of the investigational group at 24 months (+67.1 cm^3^, 95% CI [+9.1; +125.2]; *p* =.024) (Table 2, Figure 4 and Figure 5). No consistent pattern of significant differences was identified for WM volume.

Regarding the reference group, longitudinal analyses adjusted for the described propensity score indicated no statistically significant differences between the investigational and the non-randomized breastfed reference group for WM volume, but a significant slope difference between the two groups was found for the total GM volume (LSMean: +19,595 mm^3^/year in the investigational group, *p* = 0.017).

#### 3.3.4. Structural and Functional Connectivity (Secondary Outcomes)

DTI metrics (FA, RD, AD, MD) did not significantly differ between intervention groups. Insufficient high-quality matched rsfMRI data with high-resolution anatomical images were available to perform robust age-wise or longitudinal functional connectivity, and thus, results are not reported here.

Longitudinal analyses adjusted for the described propensity score indicated no statistically significant differences between the investigational and the non-randomized breastfed reference group for the DTI metrics FA, RA, and MD. For cerebellar AD, a significant slope difference was observed between the investigational and the breastfed reference group (LSMean: +0.00010 in the investigational group, *p* = 0.015).

### 3.4. Cognitive and Behavioral Results (Secondary Outcomes)

**Figure 7:**
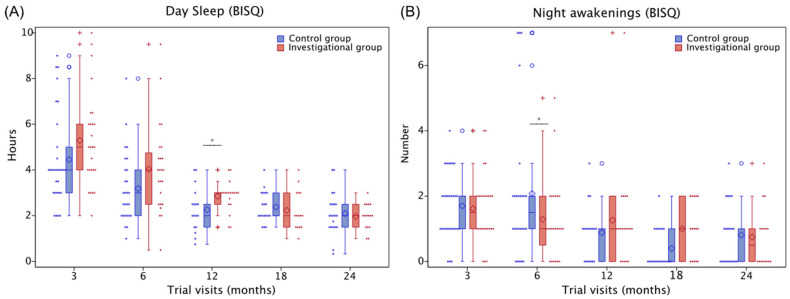
Boxplots for day sleep hours (**A**) and number of night awakenings (**B**), as assessed using the Brief Infant Sleep Questionnaire (BISQ). Day sleep hours were analyzed using the longitudinal linear mixed model, adjusted for gender and site, and night awakenings using the longitudinal negative binomial model, adjusted for gender and site. Diamonds represent the mean and horizontal lines the quartiles. Whiskers represent minimum and maximum observations within 1.5 times the interquartile range above and below the box. Dots and Crosses represent individual values. * *p* ≤ 0.05.

**Figure 8:**
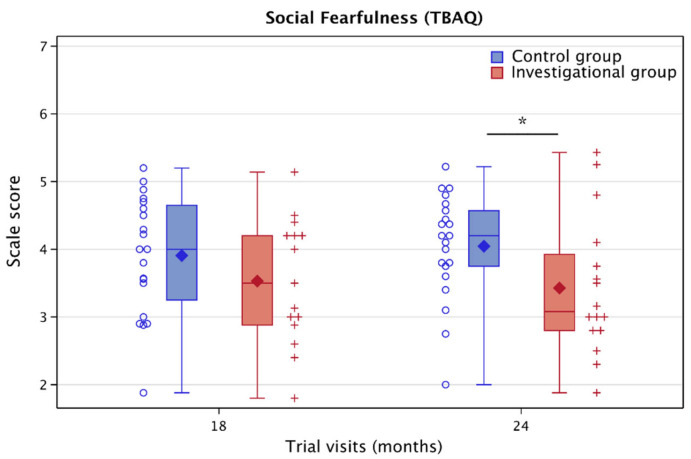
Boxplot for the social fearfulness subscale from the Toddler Behavior Assessment Questionnaire (TBAQ), analyzed using the linear mixed model, adjusted for gender and site. Diamonds represent the mean and horizontal lines the quartiles. Whiskers represent minimum and maximum observations within 1.5 times the interquartile range above and below the box. Dots and Crosses represent individual values. * *p* ≤ 0.05.

The investigational group showed a significant reduction in night awakenings at 6 months compared to the control group (odds ratio: 0.6, 95% CI [−0.36; 0.99], *p* = 0.047) and an increase in day sleep at 12 months (LSMean: +0.53 h, 95% CI [0.01; 1.04], *p* = 0.044; Figure 7) as well as a significant decrease in the social fearfulness subscale of the TBAQ at 24 months (LSMean: −0.48 points, 95% CI [−1.21; −0.013], *p* = 0.046; Figure 8).

No significant differences were found for neurodevelopment (Bayley-III cognitive, language, motor scores), overall social-emotional development (ASQ-SE2), infant behavior (IBQ-R), and performance tasks (ORTi, TapTap).

### 3.5. Correlations between Myelin and Developmental Outcomes (Exploratory Analyses)

#### 3.5.1. Motor Development

Consistent and statistically significant positive Spearman correlations were identified between the MWF (3, 6, and 12 months) and Bayley-III motor development (12 months). Correlation estimates were 0.476 (21 pairs, unadjusted *p* = 0.030), 0.619 (15 pairs, unadjusted *p* = 0.010), and 0.304 (10 pairs, unadjusted *p* = 0.390).

From the linear regression adjusted for stratification factors, it was estimated that a 0.001 MWF unit increase at 3 months was associated with a +2.25 Bayley-III motor development point increase at 12 months (95% CI [0.19; 4.32]), at 6 months was associated with a +0.92 Bayley-III motor development point increase at 12 months (95% CI [0.25; 1.6]), and at 12 months was associated with a +0.23 Bayley-III motor development point increase at 12 months (95% CI [−0.54; 1.00]).

#### 3.5.2. Language and Cognitive Development

No consistent or significant associations were identified between MRI parameters and Bayley-III language and cognition scores, TapTap, and ORTi parameters. Some positive correlations were observed between TapTap quantity of clicked objectives and GM volume, but the same parameter was negatively correlated with the MWF. Some positive non-significant correlations were observed between the ORTi reversal search strategy score, but the number of pairs of observation (≤5) prevented drawing strong conclusions about trends.

#### 3.5.3. Sleep

Consistent and statistically significant (on unadjusted *p*-values) positive Spearman correlations were identified between the MWF (3, 6, 12, 18, and 24 months), GM volume (3, 6, 12, 18, and 24 months), and BISQ night sleep (3, 6, 12, 18, and 24 months). Statistically significant correlations were estimated between GM volume at 12 months and night sleep at 18 months (+0.548, 15 pairs, unadjusted *p* = 0.034), between GM volume at 18 months and night sleep at 24 months (+0.771, 13 pairs, unadjusted *p* = 0.002), and between the MWF at 6 months and night sleep at 18 months (+0.686, 12 pairs, unadjusted *p* = 0.014). From the linear regression adjusted for stratification factors, it was estimated that a 0.001 MWF unit increase at 6 months was associated with a 0.14 h night sleep increase at 18 months (95% CI [−0.02; 0.3]); in addition, a +10 cm^3^ increase in GM volume at 12 months was associated with a +0.14 h night sleep increase at 18 months (95% CI [−0.1; 0.38]) and at 18 months was associated with a +0.15 h night sleep increase at 24 months (95% CI [0.00; 0.31]).

#### 3.5.4. Toddler Behaviors

Consistent positive Spearman correlations were identified between the MWF in the whole brain (3, 6, 12, 18, and 24 months), GM volume (3, 6, 12, 18, and 24 months), and the TBAQ social fear dimension (18 and 24 months) as well as the TBAQ activity level (18 and 24 months). Non-statistically significant negative Spearman correlations were identified between the MWF whole brain (3, 6, 12, 18, and 24 months), GM volume (3, 6, 12, 18, and 24 months), and the TBAQ anger level (18 and 24 months). Although not systematically statistically significant, the correlations were consistent across time points (Supplementary Table S3).

### 3.6. Descriptive Results (Exploratory Outcomes)

#### 3.6.1. Physical Growth and Body Composition

To evaluate the appropriateness of the children’s physical growth, the frequency of children below, within, and above the 10–90% range was calculated for weight-for-age, height-for-age, and head-circumference-for-age Z-scores according to the WHO standard. No differences were found between the intervention groups. A tendency toward higher weight-for-age Z-scores was observed at 12, 18, and 24 months of age in the formula-fed groups compared to the breastfed reference group.

PeaPod data were mostly available up to 3 months of age, and the number of available measures at 6 months dropped below 20%. Fat mass increased in all three groups from 17.1 (control group), 17.5 (investigational group), and 17.5% (breastfed group) at enrollment to 21.3 (control group), 29.4 (investigational group), and 24.3% (breastfed group) at 6 months. Fat-free mass decreased from 82.9 (control group) and 82.5% (investigational and breastfed groups) at enrollment to 78.7 (control group), 70.6 (investigational group), and 75.7% (breastfed group) at 6 months (Supplementary Table S4).

#### 3.6.2. Child Activity Level

FitBit measures for sedentary and light activity levels appeared to be the most feasible measures in the study population, while fairly and very active levels up to 18 months showed mostly a median of 0. Sedentary levels decreased in all three groups over time, from 1393 (control group), 1375 (investigational group), and 1360 (breastfed group) min at 6 months to 1252 (control group), 1244 (investigational group), and 1244 (breastfed group) min at 24 months. Light activity levels increased across the study period from 42 (control group), 63 (investigational group), and 70 (breastfed group) min at 6 months to 166 (control group), 189 (investigational group), and 176 (breastfed group) min at 24 months. We observed a trend toward being less sedentary and more lightly active in the investigational group compared to the control and breastfed reference groups at 24 months (Supplementary Table S5).

#### 3.6.3. Maternal Postnatal Depression, Parenting Stress, and Intellectual Ability

No group differences were observed for maternal postnatal depression at 6 weeks and 3 months. Overall, >90% of the mothers scored <10, the cut-off score for possible depressive symptoms.

The previously reported [14] initial tendency toward lower parenting stress scores up to 6 months in the investigational group did not continue up to 24 months. Caretakers in the breastfed reference group had a tendency of higher defensive responding and distress.

Maternal IQ scores were comparable between the intervention groups and higher for the breastfed reference group.

#### 3.6.4. Nutrient Intake

Between 15 and 20% of the infants reportedly received at least once over the study visits something other than the assigned formula. The cumulative duration of other diets was low across the 12-month intervention period and varied from 7 to 20 days. Energy intake increased in all three groups over time, from 761 (control group), 695 (investigational group), and 524 (breastfed group) kcal/day at 6 months to 1141 (control group), 1212 (investigational group), and 1196 (breastfed group) kcal/day at 24 months. The positive correlations between nutrient intake levels for the investigated blend nutrients from food intake outside of the intervention products with myelin were consistent with the causal observations for the blend impact (Supplementary Table S6). Most significant correlations were found for iron intake levels at 6 and 9 months with myelination in the first year of life, for ARA intake levels at 12 and 24 months with myelination in the second year of life, and DHA intake levels at 24 months with myelination at 24 months.

## 4. Discussion

These clinical trial findings, in addition to the previously reported 3- and 6-month data [14], are the complete results extending the initial findings up to 2 years of life. This is the first intervention trial to demonstrate the longitudinal impact of a nutrient blend on developmental myelination in children. Our hypotheses that 12-month supplementation with a blend of DHA, ARA, iron, vitamin B12, folic acid, and sphingomyelin from a uniquely processed whey protein concentrate enriched in alpha-lactalbumin and phospholipids increases myelination over the first 2 years of life in neurotypical term-born children was confirmed. Additionally, we found a significant increase in gray matter volume at 2 years for children receiving higher levels of myelin-relevant nutrients compared to the control group, as well as a significant impact of the nutrient blend on aspects of sleep and sociability. Correlational analyses between myelin, gray matter, and developmental outcomes strengthen the observations on sleep effects and demonstrate a link between first-year myelination and motor development at 1 year of life.

Myelination is a hallmark of neurodevelopment and critical for information processing, cell communication, and brain plasticity important for learning and development [15,28,29]. Our findings add to the previous observations displaying a steep increase in myelin during, particularly, the first 2 to 3 years of life [16] by showing that a specific blend of nutrients can support that trajectory. The increases observed in this study were within the MWF ranges of the non-randomized breastfed reference group and were particularly enhanced in the investigational compared to the control group not only for the whole brain (26%), frontal (32%), temporal (30%), and parietal (23%) white matter but also for the cerebellum (14%) and occipital (13%) white matter at 2 years of age. These regions are functionally relevant for several developmental domains. Frontal lobe maturation is typically linked to executive and control functions, like working memory and inhibitory control [30], as well as to voluntary movement, like walking, and associative learning [31]. It is one of the later maturing regions of the brain, with a protracted trajectory. However, the 32% difference to the control group and the enhanced growth rate may indicate a sensitive window of frontal lobe development at 2 years of age, which precedes the emergence and rapid improvements in behavioral precursors of executive functions, like inhibitory control at around age 3 [32]. In addition, white matter myelination in frontal and temporal regions has been reported to be associated with increasing expressive and receptive language abilities over the first 4 years of life [15,33]. The parietal lobe processes sensory information and is part of the efferent arm of the motor system [34]. The observed differences in myelin in that area between the intervention group and the control group could therefore lay the foundation for potential later differences in somatosensory or motor abilities, in line with the observed positive correlations between myelin and motor development. The available literature supports the link between myelination in typically developing populations across the age span with various aspects of processing speed [35,36], language development and vocabulary acquisition [33,37], reading ability [38,39], sensory reactivity and processing speed [40], working memory [41], learning and memory [16,42], and general cognitive and intellectual ability [16,42]. The absence of a significant effect on corpus callosum myelination may be linked to the slower rate of development in that area during that stage of development. The findings appear in line with an observational study in 2–8-year-old children using DTI that similarly showed an increase in fractional anisotropy (FA) across the 6-year age range, with the splenium displaying the smallest increase [43].

Gray matter volume develops rapidly in early childhood. A longitudinal observational study showed that cortical gray matter volume increases by 106% in the first year of life and 18% in the second year of life, with association cortices growing particularly rapidly in the first year and frontal and parietal regions growing quickly in the second year of life [44]. Our findings showed an impact of the nutrient blend on longitudinal gray matter development, with a significant effect in the investigational group compared to the control group at 2 years of life. While the nutrient hypothesis was built on mechanistic and observational findings related to myelination, it is possible that other brain structures and processes can be positively impacted. Gray matter contains neurons and synapses, and while neurogenesis is largely completed, synaptogenesis and synaptic plasticity remain active processes of brain development [45]. DHA as well as polar lipids have been linked to synaptic plasticity, synaptic signaling, and neurotransmission in early brain development [46,47,48]. It is therefore plausible that the investigated nutrient blend impacts brain development and connectivity through myelination as well as gray matter volume changes. Gray matter volume has been associated with, e.g., vocabulary, reading, working memory, processing speed, and set-shifting skills [49,50]. Cerebellar gray matter is described as a robust predictor of cognitive performance in children [49]. In adults, gray matter volume is linked to general cognitive ability [51].

The behavioral impact of the nutritional intervention in this study was most consistent for sleep outcomes and aspects of sociability, which are age-appropriate development domains in children of that age. Sleep behaviors mature rapidly in the first year of life, and a lower number of night awakenings as well as longer daytime naps in infants have been linked to better memory performance [52]. Social fear, an aspect of sociability linked to hesitation, distress, or shyness in novel social situations like meeting strangers, is part of normal development. Increased social fear during toddlerhood has, however, been associated with a higher risk for anxiety behaviors in later childhood [53,54]. The significant associations between myelin and motor development at 12 months may be linked to the rapid increase in motor abilities at that age [55], including the achievement of critical motor milestones like standing alone and walking without support [56,57]. The role of myelination in motor learning has also been well established in preclinical studies [58,59,60]. The 2-year age window for our study may have been too short to detect meaningful intervention-driven group differences in cognitive abilities, which might become more feasible in older children. Furthermore, behavioral measures often show a higher intra- and inter-person variability than neuroimaging measures, which may provide a methodological explanation for the findings. In our previous publication [14], we reported a trend of reduced number of night awakenings based on cross-sectional analyses with individual ANCOVAs for the two time points (3 and 6 months) available at the time. Here, we analyzed all time points longitudinally using a linear mixed model adjusted for the same covariates (sex and site), which resulted in a slight modification to the estimates, standard errors, and degrees of freedom, reaching statistical significance. The difference in statistical analyses was linked to the available two- versus multiple-time-point data and showed a consistent direction of the effect in both analyses.

Our study shows that myelin imaging is a relevant and objective marker for brain growth and associated functional outcomes in a preverbal population of neurotypical children. Our findings are in line with the suggested age-specific relationship between structure and cognition [15] as well as the reported latency between brain structural and behavioral maturation [15,16,17]. They furthermore support the notion of windows of sensitivity for specific areas of development that may benefit from age- and developmental-stage-appropriate nutritional interventions.

Strengths of the study include the longitudinal RCT design, including objective measures of brain development from neuroimaging, which strengthens nutritional research in early life. Myelin imaging has been shown to be a viable and relevant proxy for brain development that can be used to assess the impact of nutrition on brain structure and architecture. For future RCTs, it is desirable to further reduce the brain imaging protocol; additional sequences on structural and functional connectivity did not result in sufficient analyzable data in our study population, and the myelin imaging sequence was most successful in that age group.

Limitations include the impact of the COVID-19 pandemic on recruitment and retention, resulting in a smaller sample size than expected, in particular for later visits. The low sample size impacted the analyses and conclusion on intervention effects related to cognitive and behavioral outcomes. In addition, the investigational exploratory nature of the study requires cautious interpretation of the inferential statistics, as type I and type II errors are uncontrolled. To mitigate this, the number of computed *p*-values was kept to a minimum. As we did not collect the volume of intake information for the investigational groups, no conclusion on the impact of feeding schedule differences could be made in this study. However, based on the tins collected by the study sites, no differences in the used formula were apparent between the groups.

Overall, our findings add important insights into early brain architecture and behavioral development in a population where nutritional intervention studies are still scarce. They highlight an opportunity to improve developmental (i.e., de novo) myelination, a critical process in learning and development, as well as gray matter development and aspects of sleep and sociability in healthy, well-nourished infants via a nutritional intervention with levels and combinations of nutrients that were selected based on scientific evidence of their role in myelination.

## Figures and Tables

**Table 1:** Baseline characteristics of enrolled participants.

	Intervention Group		Reference Group
Characteristics	Investigational (N = 39)	Control (N = 42)	Breastfed (N = 108)
Children			
Age at enrollment (days), mean (SD)	31.6 (7.84) N_available_ = 39	31.3 (6.06) N_available_ = 42	28.7 (7.64) N_available_ = 108
Gestational age (weeks), mean (SD)	38.8 (1.24) N_available_ = 39	39.1 (1.16) N_available_ = 42	39.3 (1.11) N_available_ = 108
Female sex, number (%)	18 (46.2%) N_available_ = 39	18 (42.9%) N_available_ = 42	61 (56.5%) N_available_ = 108
Weight at birth (kg), mean (SD)	3.27 (0.45) N_available_ = 39	3.27 (0.42) N_available_ = 42	3.43 (0.46) N_available_ = 108
Height at birth (cm), mean (SD)	49.89 (2.61) N_available_ = 37	50.19 (2.31) N_available_ = 39	50.37 (2.19) N_available_ = 104
Body fat (%), mean (SD)	17.87 (5.18) N_available_ = 39	17.32 (4.74) N_available_ = 40	17.46 (4.49) N_available_ = 107
Number of siblings in same household 0 1–2 >3	14 (35.9%) 22 (56.4%) 3 (7.7%) N_available_ = 39	16 (38.1%) 22 (52.4%) 4 (9.5%) N_available_ = 42	39 (36.1%) 59 (54.6%) 10 (9.3%) N_available_ = 108
Primary Caregivers			
Ethnicity of mother	African American 5 (12.8%) Asian 1 (2.6%) Caucasian 22 (56.4%) Mixed race 2 (5.1%) Other 9 (23.1%)	African American 6 (14.3%) Caucasian 21 (50.0%) Mixed 5 (11.9%) Native American, Alaskan Native 2 (4.8%) Other 6 (14.3%)	African American 8 (7.4%) Asian 3 (2.8%) Caucasian 76 (70.4%) Mixed race 8 (7.4%) Native American, Alaskan Native 1 (0.9%) Other 12 (11.1%)
	N_available_ = 39	N_available_ = 40	N_available_ = 108
Age (years), mean (SD)			
Mother	28.7 (5.58) N_available_ = 39	28.2 (4.95) N_available_ = 40	31.6 (4.84) N_available_ = 108
Father	31.6 (6.95) N_available_ = 33	29.9 (7.31) N_available_ = 28	33.5 (5.52) N_available_ = 99
Maternal postnatal depression score at enrollment (screening visit), mean (SD)	4.2 (3.74) N_available_ = 25	6.0 (4.47) N_available_ = 26	4.4 (4.09) N_available_ = 76
Maternal IQ, mean (SD)	96.4 (10.07) N_available_ = 39	97.0 (12.47) N_available_ = 41	104.1 (13.69) N_available_ = 108

**Table 2:** Mixed model results for myelin and gray matter volume comparisons between investigational and control groups.

Region of Interest	Visit	LSMeans Difference Estimate	95% Confidence Interval	*p*-Value	Percentage (%) Increase from Control Level
Myelin Water Fraction					
Whole brain	3 months	0.001	(−0.0032; 0.0057)	0.586	3.9
	6 months	0.007	(0.0012; 0.0126)	0.018 *	22.8
	12 months	0.012	(0.0045; 0.0189)	0.002 *	22.5
	18 months	0.012	(0.0052; 0.018)	<0.001 *	18.1
	24 months	0.018	(0.0086; 0.0264)	<0.001 *	26.3
Cerebellum	3 months	0.006	(−0.001; 0.0121)	0.098	15.8
	6 months	0.01	(0.0019; 0.0187)	0.017 *	18.1
	12 months	0.018	(0.0072; 0.0286)	0.001 *	23.8
	18 months	0.011	(0.0012; 0.02)	0.028 *	11.9
	24 months	0.014	(0.001; 0.0274)	0.035 *	14.3
Corpus callosum body	3 months	0.001	(−0.0063; 0.0093)	0.706	956.5
	6 months	0.01	(0.0002; 0.0201)	0.045 *	49.0
	12 months	0.009	(−0.0038; 0.0215)	0.168	10.8
	18 months	−0.001	(−0.0121; 0.0103)	0.875	−0.9
	24 months	−0.003	(−0.019; 0.0122)	0.669	−2.4
Corpus callosum genu	3 months	0	(−0.01; 0.0109)	0.931	0
	6 months	0.011	(−0.002; 0.0245)	0.096	68.2
	12 months	0.006	(−0.0107; 0.0231)	0.464	6.1
	18 months	−0.002	(−0.0168; 0.013)	0.803	−1.5
	24 months	0.009	(−0.0118; 0.0299)	0.390	6.4
Corpus callosum splenium	3 months	0.001	(−0.0087; 0.0117)	0.772	52.5
	6 months	0.01	(−0.0028; 0.0232)	0.123	24.1
	12 months	0.008	(−0.008; 0.025)	0.308	6.8
	18 months	0.002	(−0.0121; 0.0171)	0.734	1.4
	24 months	0.002	(−0.0183; 0.0224)	0.841	1.3
Frontal white matter	3 months	0.001	(−0.005; 0.0066)	0.786	5.0
	6 months	0.007	(−0.0008; 0.0141)	0.078	29.0
	12 months	0.01	(0.0004; 0.0194)	0.041 *	19.2
	18 months	0.012	(0.0033; 0.0201)	0.007 *	17.6
	24 months	0.022	(0.01; 0.0333)	<0.001 *	31.8
Occipital white matter	3 months	0.004	(−0.0022; 0.011)	0.187	22.4
	6 months	0.011	(0.003; 0.0198)	0.009 *	32.6
	12 months	0.015	(0.0041; 0.0255)	0.007 *	24.1
	18 months	0.015	(0.0055; 0.0244)	0.002 *	19.7
	24 months	0.011	(−0.0022; 0.0241)	0.102	12.6
Parietal white matter	3 months	0.002	(−0.0033; 0.0072)	0.452	10.6
	6 months	0.008	(0.0016; 0.0151)	0.015 *	30.1
	12 months	0.015	(0.0065; 0.0235)	<0.001 *	27.0
	18 months	0.012	(0.004; 0.0191)	0.003 *	16.4
	24 months	0.018	(0.0077; 0.0287)	<0.001 *	23.3
Temporal white matter	3 months	0.002	(−0.0034; 0.008)	0.429	10.8
	6 months	0.007	(0.0002; 0.0147)	0.045 *	29.3
	12 months	0.017	(0.0079; 0.0264)	<0.001 *	34.5
	18 months	0.013	(0.0044; 0.0207)	0.003 *	19.1
	24 months	0.021	(0.0097; 0.0324)	<0.001 *	30.0
Gray Matter Volume					
Whole brain	3 months	5521.958	(−18,011.073; 29,054.9883)	0.642	1.9
	6 months	10,637.822	(−17,900.7774; 39,176.422)	0.461	2.9
	12 months	23,221.587	(−9471.0578; 55,914.231)	0.162	5.2
	18 months	19,424.425	(−16,330.1755; 55,179.0258)	0.283	4.0
	24 months	67,147.449	(9087.0565; 125,207.8421)	0.024 *	13.1

* *p* ≤ 0.05; LSMeans = least square means.

## Data Availability

Datasets are available on request, without undue reservation. Deidentified individual participant data will not be made available.

## Supplementary Materials

The following supporting information can be downloaded at: https://www.mdpi.com/article/10.3390/nu15204439/s1, Table S1. Summary of MRI acquisition protocols; Table S2. Feeding characteristics for study groups; Table S3. Spearman correlations between myelin, gray matter volume and social-emotional subscales of TBAQ (social fear, activity level, anger); Table S4. Descriptive PeaPod body composition data for fat mass and fat-free mass; Table S5. Descriptive activity level data from FitBit; Table S6. Correlations between nutrient intake levels and myelin.

## Clinical Trial Registration

https://clinicaltrials.gov/study/NCT03111927, accessed on 19 June 2019, identifier: NCT03111927 (Infant Nutrition and Brain Development-Full Text View-FClinicalTrials.gov).
