# Supplementary material for: Impact of a Nutrient Formulation on Longitudinal Myelination, Cognition, and Behavior from Birth to 2 Years: A Randomized Clinical Trial
Source: Nutrients. 2023 Oct 19;15(20):4439. doi: 10.3390/nu15204439 (PMC10610069; doi:10.3390/nu15204439)
Supplement: Supplementary file 1 [file nutrients-15-04439-s001.zip › nutrients-2630884-supplementary.pdf]

Supplementary Material

Impact of a Nutrient Formulation on Longitudinal Myelination, Cognition, and Behavior from Birth to 2 Years: A Randomized Clinical Trial

Nora Schneider[1*], Mickaël Hartweg[2], Jonathan O'Regan[3], Jennifer Beauchemin[4], Leanne Redman[5], Daniel S. Hsia[5], Pascal Steiner[1], Owen Carmichael[5], Viren D'Sa[4,6], Sean C. L. Deoni[6,7]

[1]Brain Health, Nestlé Institute of Health Sciences, Nestlé Research, Société des Produits Nestlé SA, Lausanne, Switzerland

[2]Biostatistics and Data Management, Clinical Research Unit, Nestlé Research, Société des Produits Nestlé SA, Vers-chez-les-Blanc, Lausanne, Switzerland

[3]Nestlé Development Centre Nutrition, Askeaton, Co. Limerick, Ireland

[4]Advanced Baby Imaging Lab, Hasbro Children's Hospital, Providence, USA

[5]Pennington Biomedical Research Center, Baton Rouge, Louisiana, USA

[6] Department of Pediatrics, Brown University, Providence, USA

[7] Spinn Neuroscience, 98275 Mukilteo, WA

*** Correspondence:** Nora Schneider: nora.schneider@rdls.nestle.com

Supplementary Figures and Tables

Supplementary Table S1. Summary of MRI acquisition protocols

Sequence		Parameter	3 & 6 Months	12 & 18 Months	24 Months
mcDESPOT	SPGR	FOV (cm^2)	16 x 16	19 x 19	22 x 22
		Matrix	96 x 96	112 x 112	128 x 128
		# Slices	80	84	92
		TE/TR (ms)	5.8 / 14	5.4 / 14	5.4 / 14
		Flip Angles	2, 3, 4, 5, 6, 7, 10, 14	2, 3, 4, 5, 6, 7, 10, 14	2, 3, 4, 5, 6, 7, 10, 14
		Bandwidth	350 Hz/Pixel	350 Hz/Pixel	350 Hz/Pixel
	IR-SPGR	FOV (cm^2)	16 x 16	19 x 19	22 x 22
		Matrix	64 x 64	64 x 64	64 x 64
		# Slices	40	42	46
		TE/TR (ms)	5.8 / 14	5.4 / 14	5.3 / 14
		TI (ms)	950	950	950
		Flip Angles	5	5	5
		Bandwidth	350 Hz/Pixel	350 Hz/Pixel	350 Hz/Pixel
	bSSFP	FOV (cm^2)	16 x 16	19 x 19	22 x 22
		Matrix	96 x 96	112 x 112	128 x 128
		# Slices	80	84	92
		TE/TR (ms)	6 / 12	6 / 12	6 / 12
		Flip Angles	12, 16, 19, 23, 27, 35, 50, 70	12, 16, 19, 23, 27, 35, 50, 70	12, 16, 19, 23, 27, 35, 50, 70
		Bandwidth	350 Hz/Pixel	350 Hz/Pixel	350 Hz/Pixel
MP-RAGE		FOV (cm^2)	16 x 16	19 x 19	22 x 22
		Matrix	64 x 64	64 x 64	64 x 64
		# Slices	40	42	46
		TE/TR (ms)	5.8 / 14	5.4 / 14	5.3 / 14
		TI (ms)	950	950	950
		Flip Angle	5	5	5
		Bandwidth	350 Hz/Pixel	350 Hz/Pixel	350 Hz/Pixel
DTI		FOV (cm^2)	16 x 16	19 x 19	22 x 22
		Matrix	64 x 64	64 x 64	64 x 64
		# Slices	48	48	48
		Slice Thickness (mm)	2.5	2.5	2.5
		Orientation	Axial	Axial	Axial
		TE/TR (ms)	104 / 6700	104 / 6700	104 / 6700
		B-Values (s/mm^2)	0, 700, 1500, 2000	0, 700, 1500, 2000	0, 700, 1500, 2000
		# Diffusion Directions	2 / 12 / 30 / 30	2 / 12 / 30 / 30	2 / 12 / 30 / 30
		Acceleration Factor	2	2	2
rsfMRI		FOV (cm^2)	16 x 16	19 x 19	22 x 22
		Matrix	64 x 64	64 x 64	64 x 64

	# Slices	42	42	42
	Slice Thickness (mm)	3.6	3.6	3.6
	Orientation	Axial	Axial	Axial
	TE/TR (ms)	34 / 2500	34 / 2500	34 / 2500
	Flip Angle	80	80	80
	# Volumes	164	164	164
	Acceleration Factor	2	2	2

Supplementary Table S2. Feeding characteristics for study groups

Feeding information		Visit	Control group (n=42)	Investigational group (n=39)	Breast-fed group (n=108)
Number of feeds per day over the past 7 days, Mean (SD)		6 weeks	6.9 (2.9)	8.1 (2.3)	9 (2.47)
		3 months	5.7 (2.6)	6.9 (2.9)	8 (2.3)
		6 months	5.3 (2.1)	4.7 (1.9)	6.6 (2.4)
		9 months	5 (1.9)	4.5 (1.4)	5.6 (2.4)
		12 months	2.2 (2.1)	3.1 (2.3)	4.4 (1.9)
Feeding mode, n (%)	No response	6 weeks	2 (5.1)	3 (7.1)	9 (8.3)
	On a fixed schedule		1 (2.6)	4 (9.5)	4 (3.7)
	On demand		25 (64.1)	24 (57.1)	2 (1.9)
	On demand and on a fix schedule		4 (10.3)	4 (9.5)	93 (86.1)
	No response	3 months	6 (15.4)	4 (9.5)	31 (28.7)
	On a fixed schedule		1 (2.6)	4 (9.5)	6 (5.6)
	On demand		19 (48.7)	24 (57.1)	3 (2.8)
	On demand and on a fix schedule		6 (15.4)	3 (7.1)	68 (63.0)
	No response	6 months	10 (25.6)	9 (21.4)	44 (40.7)
	On a fixed schedule		5 (12.8)	7 (16.7)	10 (9.3)
	On demand		13 (33.3)	16 (38.1)	9 (8.3)
	On demand and on a fix schedule		4 (10.3)	3 (7.1)	45 (41.7)
	No response	9 months	12 (30.8)	9 (21.4)	59 (54.6)
	On a fixed schedule		3 (7.7)	9 (21.4)	7 (6.5)
	On demand		12 (30.8)	12 (28.6)	6 (5.6)
	On demand and on a fix schedule		5 (12.8)	5 (11.9)	36 (33.3)
	No response	12 months	13 (33.3)	16 (38.1)	67 (62.0)
	On a fixed schedule		6 (15.4)	12 (28.6)	10 (9.3)
	On demand		10 (25.6)	5 (11.9)	11 (10.2)
	On demand and on a fix schedule		3 (7.7)	2 (4.8)	20 (18.5)
Encouragement to finish a bottle (expressed milk for breastfeeding group) if baby stops drinking, n (%)	No response	6 weeks	2 (5.1)	3 (7.1)	104 (96.3)
	No		20 (51.3)	22 (52.4)	3 (2.8)
	Yes		10 (25.6)	10 (23.8)	1 (0.9)
	No response	3 months	6 (15.4)	4 (9.5)	102 (94.4)
	No		14 (35.9)	22 (52.4)	5 (4.6)
	Yes		12 (30.8)	9 (21.4)	1 (0.9)
	No response	6 months	10 (25.6)	9 (21.4)	102 (94.4)
	No		13 (33.3)	21 (50.0)	5 (4.6)
	Yes		9 (23.1)	5 (11.9)	1 (0.9)
	No response	9 months	12 (30.8)	9 (21.4)	103 (95.4)
	No		12 (30.8)	20 (47.6)	2 (1.9)
	Yes		8 (20.5)	6 (14.3)	3 (2.8)
	No response	12 months	13 (33.3)	16 (38.1)	105 (97.2)
	No		13 (33.3)	15 (35.7)	2 (1.9)
	Yes		6 (15.4)	4 (9.5)	1 (0.9)
Number of infants with at least one reported interruption of study product intake/ breastfeeding, n (%)	No response	6 weeks	2 (5.1)	3 (7.1)	9 (8.3)
	No		28 (71.8)	32 (76.2)	98 (90.7)
	Yes		2 (5.1)	0	1 (0.9)
	No response	3 months	9 (23.1)	4 (9.5)	30 (27.8)
	No		21 (53.8)	28 (66.7)	76 (70.4)
	Yes		2 (5.1)	3 (7.1)	2 (1.9)
	No response	6 months	10 (25.6)	8 (19)	46 (42.6)
	No		22 (56.4)	25 (59.5)	61 (56.5)
	Yes		0	2 (4.8)	1 (0.9)
	No response	9 months	12 (30.8)	9 (21.4)	60 (55.6)
	No		20 (51.3)	26 (61.9)	48 (44.4)

Feeding information		Visit	Control group (n=42)	Investigational group (n=39)	Breast-fed group (n=108)
	Yes		0	0	0
	No response	12 months	17 (43.6)	18 (42.9)	67 (62.0)
	No		15 (38.5)	15 (35.7)	41 (38.0)
	Yes		0	2 (4.8)	9 (8.3)
Length of interruption (days), Mean (SD)		6 weeks	0.5 (2.0)	0 (0)	0 (0.3)
		3 months	0.8 (3.3)	2.5 (10.9)	0.1 (0.3)
		6 months	0 (0)	2.4 (11.7)	0.1 (1.0)
		9 months	0 (0)	0 (0)	0 (0)
		12 months	0 (0)	0.6 (2.0)	0 (0)
Other dietary intake than the assigned study formula, n (%)	Missing	6 weeks	2 (5.1)	3 (7.1)	N/A
	No		22 (56.4)	23 (54.8)	N/A
	Yes		8 (20.5)	9 (21.4)	N/A
	Missing	3 months	6 (15.4)	3 (7.1)	N/A
	No		20 (51.3)	26 (61.9)	N/A
	Yes		6 (15.4)	6 (14.3)	N/A
	Missing	6 months	8 (20.5)	7 (16.7)	N/A
	No		22 (56.4)	22 (52.4)	N/A
	Yes		2 (5.1)	6 (14.3)	N/A
Cumulative duration of other dietary intake (days), Mean (SD)		6 weeks	7.5 (14.9)	7 (14.3)	N/A
		3 months	1.5 (4.1)	5.5 (14.5)	N/A
		6 months	7.7 (33.0)	19.1 (45.9)	N/A

Note: n (%) refers the number of participants with at least one interruption, divided by the number of participants in the group, and multiplied by 100. Participants that missed the visit are not counted.

Supplementary Table S3. Spearman correlations between myelin, gray matter volume and social-emotional subscales of TBAQ (social fear, activity level, anger)

Parameter 1	Timepoint 1	Parameter 2	Timepoint 2	Spearman correlation	P value	Number of pairs
Gray Matter Volume	3 months	Activity level	18 months	0.005	0.983	25
			24 months	-0.051	0.811	25
		Anger	18 months	0.096	0.647	25
			24 moths	-0.169	0.419	25
		Social Fear	18 months	-0.002	0.991	25
			24 months	0.05	0.811	25
	6 months	Activity level	18 months	0.219	0.382	18
			24 months	0.355	0.136	19
		Anger	18 months	0.246	0.325	18
			24 months	-0.138	0.573	19
		Social Fear	18 months	-0.043	0.864	18
			24 months	-0.082	0.739	19
	12 months	Activity level	18 months	0.054	0.837	17
			24 months	-0.005	0.985	15
		Anger	18 months	0.103	0.694	17
			24 months	0.061	0.830	15
		Social Fear	18 months	0.077	0.768	17
			24 months	0.127	0.651	15
	18 months	Activity level	18mo	0.206	0.462	15
			24mo	0.309	0.304	13
		Anger	18mo	0.136	0.629	15
			24mo	0.044	0.887	13
		Social Fear	18mo	-0.58	0.023	15
			24mo	-0.454	0.119	13
	24 months	Activity level	18mo	0.559	0.192	7
			24mo	0.679	0.093	7
		Anger	18mo	0.179	0.702	7
			24mo	0.378	0.403	7
		Social Fear	18mo	-0.393	0.383	7
			24mo	-0.179	0.702	7
Whole Brain MWF	3 months	Activity level	18mo	0.208	0.379	20
			24mo	0.141	0.553	20
		Anger	18mo	0.389	0.090	20
			24mo	0.118	0.620	20
		Social Fear	18mo	0.005	0.985	20
			24mo	-0.038	0.800	20
	6 months	Activity level	18mo	0.13	0.688	12
			24mo	-0.224	0.461	13
		Anger	18mo	0.47	0.123	12
			24mo	0.313	0.297	13
		Social Fear	18mo	0.196	0.540	12
			24mo	-0.407	0.168	13
	12 months	Activity level	18mo	0.328	0.354	10
			24mo	0.395	0.333	8
		Anger	18mo	0.444	0.199	10
			24mo	0.69	0.058	8
		Social Fear	18mo	0.158	0.663	10
			24mo	-0.738	0.037	8
	18 months	Activity level	18mo	0.442	0.099	15

Parameter 1	Timepoint 1	Parameter 2	Timepoint 2	Spearman correlation	P value	Number of pairs
			24mo	0.238	0.434	13
		Anger	18mo	0.403	0.137	15
			24mo	0.536	0.059	13
		Social Fear	18mo	-0.229	0.412	15
			24mo	-0.743	0.004	13
	24 months	Activity level	18mo	0.539	0.168	8
			24mo	0.548	0.160	8
		Anger	18mo	0.071	0.867	8
			24mo	0.405	0.320	8
		Social Fear	18mo	-0.635	0.091	8
			24mo	-0.524	0.183	8

Supplementary Table S4. Descriptive PeaPod body composition data for fat mass and fat-free mass.

		Mean		SD		Minimum		Maximum	
**Study group**	**N available**	**Kg**	**%**	**Kg**	**%**	**Kg**	**%**	**Kg**	**%**
**Fat Mass**									
Visit 0 (2 to 5 weeks of age)									
Control group	33	0.7	17.1	0.3	5.1	0.2	6.9	1.2	27.2
Investigational group	32	0.8	17.5	0.3	5.4	0.2	4.9	1.5	31.3
Breast-fed reference	107	0.7	17.5	0.2	4.5	0.1	3.0	1.5	28.7
Visit 1 (6 weeks of age +/- 1 week)									
Control group	31	0.9	19.3	0.3	5.2	0.3	9.1	1.6	28.5
Investigational group	28	1.0	21.1	0.3	3.6	0.6	14.5	1.7	28.6
Breast-fed reference	100	1.0	19.9	0.3	4.9	0.2	4.6	1.7	31.8
Visit 2 (3 months of age +/- 2 weeks)									
Control group	27	1.4	23.3	0.5	6.0	0.7	11.3	2.4	36.0
Investigational group	22	1.5	24.7	0.4	5.0	0.9	16.3	2.2	32.8
Breast-fed reference	82	1.4	24.0	0.5	5.3	0.1	13.0	2.4	35.2
Visit 3 (6 months of age +/- 2 weeks)									
Control group	6	1.5	21.3	0.8	9.8	0.6	7.7	2.5	32.1
Investigational group	5	2.0	29.4	0.4	4.2	1.4	23.2	2.4	34.1
Breast-fed reference	19	1.6	24.3	0.4	5.1	1.0	15.1	2.6	32.8
**Fat-Free Mass**									
Visit 0 (2 to 5 weeks of age)									

		Mean		SD		Minimum		Maximum	
**Study group**	**N available**	**Kg**	**%**	**Kg**	**%**	**Kg**	**%**	**Kg**	**%**
Control group	33	3.5	82.9	0.4	5.1	2.6	72.8	4.3	93.1
Investigational group	32	3.5	82.5	0.5	5.4	2.6	68.7	4.3	95.1
Breast-fed reference	107	3.4	82.5	0.4	4.5	2.5	71.3	4.6	97.0
Visit 1 (6 weeks of age +/- 1 week)									
Control group	31	3.8	80.7	0.5	5.2	2.8	71.5	4.5	90.9
Investigational group	28	3.8	78.9	0.5	3.6	2.7	71.4	4.8	85.5
Breast-fed reference	100	3.8	80.1	0.4	4.9	3.0	68.2	5.0	95.4
Visit 2 (3 months of age +/- 2 weeks)									
Control group	27	4.6	76.7	0.5	6.0	3.5	64.0	5.5	88.7
Investigational group	22	4.5	75.3	0.5	5.0	3.7	67.2	5.6	83.7
Breast-fed reference	82	4.4	76.0	0.5	5.3	3.5	64.8	5.7	87.0
Visit 3 (6 months of age +/- 2 weeks)									
Control group	6	5.4	78.7	0.8	9.8	4.7	67.9	7.0	92.3
Investigational group	5	4.9	70.6	0.5	4.2	4.3	65.9	5.6	76.8
Breast-fed reference	19	5.1	75.7	0.4	5.1	4.3	67.2	5.9	84.9

Supplementary Table S5. Descriptive activity level data from FitBit.

Sedentary (min/week)								
**Study group**	**N available**	**Mean**	**Median**	**SD**	**Q1**	**Q3**	**Minimum**	**Maximum**
Visit 3 (6 months of age +/- 2 weeks)								
Control group	11	1392.7	1392.5	42.20	1363.7	1437.7	1304.3	1440.0
Investigational group	12	1374.8	1384.8	39.79	1360.2	1400.8	1301.3	1429.0
Breast-fed reference	34	1360.0	1365.8	52.24	1340.3	1395.0	1201.3	1434.3
Visit 4 (9 months of age +/- 2 weeks)								
Control group	10	1340.7	1354.3	58.68	1280.3	1365.3	1256.3	1436.3
Investigational group	8	1328.3	1329.5	55.05	1281.7	1382.7	1252.3	1386.3
Breast-fed reference	23	1366.3	1365.3	40.95	1334.0	1405.3	1292.3	1431.0
Visit 5 (12 months of age +/- 2 weeks)								
Control group	10	1318.1	1317.7	77.24	1282.0	1366.0	1151.7	1439.3
Investigational group	10	1310.3	1305.5	53.63	1263.3	1350.7	1251.7	1394.3
Breast-fed reference	15	1314.8	1329.0	66.28	1265.3	1339.0	1212.7	1440.0
Visit 6 (18 months of age +/- 3 weeks)								
Control group	9	1254.6	1243.0	61.99	1232.3	1244.0	1196.7	1410.0
Investigational group	7	1237.4	1214.7	60.91	1184.0	1270.0	1182.7	1349.0
Breast-fed reference	13	1249.6	1269.0	55.21	1207.7	1280.3	1162.7	1339.7
Visit 7 (24 months of age +/- 4 weeks)								
Control group	7	1251.8	1258.3	39.07	1219.0	1269.0	1194.0	1315.7
Investigational group	5	1243.5	1260.7	56.33	1212.0	1282.7	1162.0	1300.3

Sedentary (min/week)								
**Study group**	**N available**	**Mean**	**Median**	**SD**	**Q1**	**Q3**	**Minimum**	**Maximum**
Breast-fed reference	6	1247.1	1235.3	112.76	1161.0	1292.7	1118.3	1440.0
**Lightly active (min/week)**								
Visit 3 (6 months of age +/- 2 weeks)								
Control group	11	42.3	47.5	35.62	2.3	62.0	0.0	116.0
Investigational group	12	63.4	55.2	39.09	39.2	71.0	11.0	138.7
Breast-fed reference	34	70.2	60.3	44.78	38.3	94.3	5.7	196.0
Visit 4 (9 months of age +/- 2 weeks)								
Control group	10	96.2	83.7	59.60	61.7	159.7	3.7	183.7
Investigational group	8	110.0	110.5	54.89	55.0	156.7	51.3	184.7
Breast-fed reference	23	72.7	74.7	40.95	34.7	106.0	9.0	147.7
Visit 5 (12 months of age +/- 2 weeks)								
Control group	10	112.8	116.2	64.14	74.0	155.7	0.7	233.3
Investigational group	10	127.5	134.5	55.72	89.3	176.7	30.3	185.0
Breast-fed reference	15	122.3	111.0	63.29	98.0	171.3	0.0	222.3
Visit 6 (18 months of age +/- 3 weeks)								
Control group	9	173.6	189.0	56.28	171.7	202.3	30.0	219.0
Investigational group	7	192.8	221.7	57.99	148.0	236.0	91.0	252.7
Breast-fed reference	13	175.8	167.7	50.66	138.0	205.7	100.3	260.0
Visit 7 (24 months of age +/- 4 weeks)								
Control group	7	165.7	161.3	36.89	143.0	183.7	115.7	232.3

Sedentary (min/week)								
Study group	N available	Mean	Median	SD	Q1	Q3	Minimum	Maximum
Investigational group	5	189.1	170.0	52.07	152.0	225.0	137.7	261.0
Breast-fed reference	6	176.6	186.7	104.32	137.7	244.7	0.0	304.0

Supplementary Table S6. Correlations between nutrient intake levels and myelin

	Number of pairs	Spearman Correlation coefficient	P-value
**Correlations with myelin water fraction (mm^3) at 6 months**			
ARA intake (g/day) at 6 months	8	0.62	0.102
DHA intake (g/day) at 6 months	8	0.62	0.102
Folic acid intake (mcg/day) at 6 months	8	0.67	0.071
Iron intake (mg/day) at 6 months	8	0.76	0.028
Sphingomyelin intake (mg/day) at 6 months	8	0.62	0.102
Vitamin B12 intake (mcg/day) at 6 months	8	0.62	0.102
**Correlations with myelin water fraction (mm^3) at 12 months**			
ARA intake (g/day) at 6 months	5	0.60	0.285
ARA intake (g/day) at 9 months	4	0.80	0.200
ARA intake (g/day) at 12 months	4	1.00	<0.001
DHA intake (g/day) at 6 months	5	0.60	0.285
DHA intake (g/day) at 9 months	4	0.80	0.200
DHA intake (g/day) at 12 months	4	0.40	0.600
Folic acid intake (mcg/day) at 6 months	5	0.60	0.285
Folic acid intake (mcg/day) at 9 months	4	0.80	0.200
Folic acid intake (mcg/day) at 12 months	4	0.20	0.800
Iron intake (mg/day) at 6 months	5	0.90	0.037
Iron intake (mg/day) at 9 months	4	1.00	<0.001
Iron intake (mg/day) at 12 months	4	0.40	0.600
Sphingomyelin intake (mg/day) at 6 months	5	0.60	0.285
Sphingomyelin intake (mg/day) at 9 months	4	0.80	0.200
Sphingomyelin intake (mg/day) at 12 months	4	0.80	0.200
Vitamin B12 intake (mcg/day) at 6 months	5	0.6	0.285
Vitamin B12 intake (mcg/day) at 9 months	4	0.8	0.200
Vitamin B12 intake (mcg/day) at 12 months	4	0.8	0.200
**Correlations with myelin water fraction (mm^3) at 18 months**			
ARA intake (g/day) at 6 months	8	0.57	0.139
ARA intake (g/day) at 9 months	6	0.66	0.156
ARA intake (g/day) at 12 months	7	0.75	0.052
ARA intake (g/day) at 18 months	8	0.14	0.736
DHA intake (g/day) at 6 months	8	0.10	0.823
DHA intake (g/day) at 9 months	6	0.66	0.156
DHA intake (g/day) at 12 months	7	0.21	0.645
DHA intake (g/day) at 18 months	8	0.00	1.000
Folic acid intake (mcg/day) at 6 months	8	0.10	0.823
Folic acid intake (mcg/day) at 9 months	6	0.37	0.468
Folic acid intake (mcg/day) at 12 months	7	-0.32	0.482
Folic acid intake (mcg/day) at 18 months	8	-0.50	0.207

	Number of pairs	Spearman Correlation coefficient	P-value
Iron intake (mg/day) at 6 months	8	0.31	0.456
Iron intake (mg/day) at 9 months	6	-0.20	0.704
Iron intake (mg/day) at 12 months	7	-0.29	0.535
Iron intake (mg/day) at 18 months	8	-0.67	0.071
Sphingomyelin intake (mg/day) at 6 months	8	0.40	0.320
Sphingomyelin intake (mg/day) at 9 months	6	0.60	0.208
Sphingomyelin intake (mg/day) at 12 months	7	0.57	0.180
Sphingomyelin intake (mg/day) at 18 months	8	0.14	0.736
Vitamin B12 intake (mcg/day) at 6 months	8	0.17	0.693
Vitamin B12 intake (mcg/day) at 9 months	6	0.37	0.469
Vitamin B12 intake (mcg/day) at 12 months	7	-0.25	0.589
Vitamin B12 intake (mcg/day) at 18 months	8	-0.33	0.420
**Correlations with myelin water fraction (mm^3) at 24 months**			
ARA intake (g/day) at 6 months	4	0.80	0.200
ARA intake (g/day) at 9 months	4	0.40	0.600
ARA intake (g/day) at 12 months	5	0.20	0.747
ARA intake (g/day) at 18 months	4	0.60	0.400
ARA intake (g/day) at 24 months	4	1.00	<0.001
DHA intake (g/day) at 6 months	4	0.80	0.200
DHA intake (g/day) at 9 months	4	0.40	0.600
DHA intake (g/day) at 12 months	5	-0.10	0.873
DHA intake (g/day) at 18 months	4	0.80	0.200
DHA intake (g/day) at 24 months	4	1.00	<0.001
Folic acid intake (mcg/day) at 6 months	4	0.80	0.200
Folic acid intake (mcg/day) at 9 months	4	0.40	0.600
Folic acid intake (mcg/day) at 12 months	5	0.00	1.000
Folic acid intake (mcg/day) at 18 months	4	-0.80	0.200
Folic acid intake (mcg/day) at 24 months	4	-0.40	0.600
Iron intake (mg/day) at 6 months	4	0.20	0.800
Iron intake (mg/day) at 9 months	4	0.40	0.600
Iron intake (mg/day) at 12 months	5	0.60	0.285
Iron intake (mg/day) at 18 months	4	-0.80	0.200
Iron intake (mg/day) at 24 months	4	0.40	0.600
Sphingomyelin intake (mg/day) at 6 months	4	0.80	0.200
Sphingomyelin intake (mg/day) at 9 months	4	0.40	0.600
Sphingomyelin intake (mg/day) at 12 months	5	-0.10	0.873
Sphingomyelin intake (mg/day) at 18 months	4	0.80	0.200
Sphingomyelin intake (mg/day) at 24 months	4	0.80	0.200
Vitamin B12 intake (mcg/day) at 6 months	4	0.80	0.200
Vitamin B12 intake (mcg/day) at 9 months	4	-0.20	0.800
Vitamin B12 intake (mcg/day) at 12 months	5	0.30	0.624
Vitamin B12 intake (mcg/day) at 18 months	4	1.00	<0.001
Vitamin B12 intake (mcg/day) at 24 months	4	0.40	0.600
